# Follistatin promotes adipocyte differentiation, browning, and energy metabolism[S]

**DOI:** 10.1194/jlr.M039719

**Published:** 2014-03

**Authors:** Melissa Braga, Srinivasa T. Reddy, Laurent Vergnes, Shehla Pervin, Victor Grijalva, David Stout, John David, Xinmin Li, Venina Tomasian, Christopher B. Reid, Keith C. Norris, Sherin U. Devaskar, Karen Reue, Rajan Singh

**Affiliations:** * Division of Endocrinology and Charles R. Drew University of Medicine and Science, Los Angeles, CA 90059; and; §§ Metabolism and Life Science Institute, Charles R. Drew University of Medicine and Science, Los Angeles, CA 90059; and; † Departments of Obstetrics and Gynecology, David Geffen School of Medicine at University of California Los Angeles, Los Angeles, CA 90095; § Medicine, David Geffen School of Medicine at University of California Los Angeles, Los Angeles, CA 90095; ** Molecular and Medical Pharmacology, David Geffen School of Medicine at University of California Los Angeles, Los Angeles, CA 90095; †† Human Genetics, David Geffen School of Medicine at University of California Los Angeles, Los Angeles, CA 90095; *** Pathology and Laboratory Medicine, and David Geffen School of Medicine at University of California Los Angeles, Los Angeles, CA 90095; ††† Pediatrics, David Geffen School of Medicine at University of California Los Angeles, Los Angeles, CA 90095

**Keywords:** mouse embryonic fibroblast, myostatin, brown fat, energy expenditure, uncoupling protein 1, mitochondria

## Abstract

Follistatin (Fst) functions to bind and neutralize the activity of members of the transforming growth factor-β superfamily. Fst has a well-established role in skeletal muscle, but we detected significant Fst expression levels in interscapular brown and subcutaneous white adipose tissue, and further investigated its role in adipocyte biology. Fst expression was induced during adipogenic differentiation of mouse brown preadipocytes and mouse embryonic fibroblasts (MEFs) as well as in cold-induced brown adipose tissue from mice. In differentiated MEFs from Fst KO mice, the induction of brown adipocyte proteins including uncoupling protein 1, PR domain containing 16, and PPAR gamma coactivator-1α was attenuated, but could be rescued by treatment with recombinant FST. Furthermore, Fst enhanced thermogenic gene expression in differentiated mouse brown adipocytes and MEF cultures from both WT and Fst KO groups, suggesting that Fst produced by adipocytes may act in a paracrine manner. Our microarray gene expression profiling of WT and Fst KO MEFs during adipogenic differentiation identified several genes implicated in lipid and energy metabolism that were significantly downregulated in Fst KO MEFs. Furthermore, Fst treatment significantly increases cellular respiration in Fst-deficient cells. Our results implicate a novel role of Fst in the induction of brown adipocyte character and regulation of energy metabolism.

Obesity has reached epidemic proportions world-wide, with more than a billion adults being overweight (defined as a BMI greater than 25 kg/m^2^), and over 300 million adults classified as obese (BMI >30 kg/m^2^) (1). Obesity is a major risk factor for diabetes, insulin resistance, dyslipidemia, nonalcoholic fatty liver, cardiovascular disease, and some cancers (2, 3). Of concern is the economic impact of obesity which continues to rise steeply. Obesity develops from an imbalance between energy intake and expenditure (1, 2). While most therapies for obesity focus on reducing caloric intake and exercise (4, 5), increasing cellular energy expenditure has been proposed as an attractive alternate approach (6, 7).

Brown adipose tissue (BAT) has a remarkable energy dissipating capacity and actively promotes triglyceride clearance, glucose disposal, and generation of heat for thermogenesis (8–10). Brown fat cells have a high mitochondrial content and the ability to uncouple cellular respiration through uncoupling protein 1 (UCP1) (9). Hence the amount of BAT activity impacts systemic energy balance, and may be a determinant of both obesity and the related dysregulation of lipid metabolism observed in the metabolic syndrome (8, 9). Transgenic mice that overexpress UCP1 are resistant to genetic and diet-induced obesity (11). It is evident from recent reports that there exist two different types of UCP1-positive adipocytes with distinctly different developmental origins. These are classical brown adipocytes, which are found in the interscapular regions, and pockets of UCP1-positive adipocytes that are found within white adipose tissue (WAT) depots (12–17). These inducible brown-like adipocytes known as “brite” (brown-within-white) or “beige” adipocytes possess many of the biological and morphological characteristics of classical brown adipocytes. Importantly, some factors that promote the “browning” of WAT have been identified, including PPARγ agonists, SirT1-dependent deacetylation of PPARγ, activation of the hypothalamic-adipocyte axis, and loss of myostatin (Mst) and neuronatin (13–17).

We recently demonstrated that brown adipocyte differentiation is inhibited by Mst, a negative regulator of muscle mass (18). In both mice and primary cell cultures, Mst inactivation promoted the expression of key brown adipocyte genes, including Ucp1, PR domain containing 16 (Prdm16), Pgc1a, and bone morphogenetic protein 7 (Bmp7) (18). Mst inactivation also protects mice from diet-induced obesity by blockade of the transforming growth factor-β (TGF-β)/Mst/Smad3 signaling cascade (19, 20). Previously, we observed that androgens upregulated the expression of follistatin (Fst) and antagonized TGF-β signaling in various cell lines (18, 21). Because Fst is known to antagonize Mst and inhibit overall TGF-β signaling, we hypothesized that Fst may promote the browning of white adipocytes. Here we demonstrate that Fst enhances the acquisition of brown adipocyte characteristics, including expression of thermogenic markers and increased respiratory function.

## MATERIALS AND METHODS

### Animals

Male and female Fst heterozygous (Fst^+/−^) mice were a kind gift from Dr. Martin Matzuk (Baylor College of Medicine) (22). Mating between heterozygous males and females was performed and embryos (∼13.5 days) were collected for preparation of WT (+/+) and KO (−/−) mouse embryonic fibroblasts (MEFs). All mice were housed at constant temperature (74°F) under an artificial light/dark cycle (12/12 h) and allowed free access to food and water. Animal experiments were approved by the Institutional Animal Care and Use Committee. For cold exposure, C57BL/6 mice were kept at 4–6°C for 8 h and BAT was harvested and compared with tissue from the room temperature mice.

### Differentiation of brown preadipocytes

Immortalized brown preadipocytes were received as a gift from Dr. Bruce Spiegelman, Harvard Medical Center (23). Confluent cells seeded on 6-well plates were exposed to an adipogenic cocktail containing 5 μM dexamethasone, 20 nM insulin, 0.5 mM isobutylmethylxanthine (IBMX), 1 nM T3, and 125 μM indomethacin (Sigma Chemicals, St. Louis, MO) in the presence or absence of 0.5 μg/ml recombinant FST protein (R&D Systems, Minneapolis, MN) (23). Forty-eight hours after adipogenic induction, culture medium was replaced with maintenance medium containing 20 nM insulin and 1 nM T3 with or without FST for an additional 6 days. Cells were harvested and analyzed for adipocyte-specific markers by Western blot and real-time quantitative PCR (qPCR) analysis.

### MEF culture, differentiation, and treatment

MEFs were generated from 13.5 day postcoitum mouse embryos as described before (18). Embryos were harvested; the head, limbs, and the internal organs were removed; and the carcasses were rinsed in 1× PBS and minced. Minced carcasses were suspended with 3 ml 0.025% trypsin/EDTA (Invitrogen, Carlsbad, CA) and incubated at 37°C for 20 min. After two trypsinization cycles, the trypsin was neutralized by adding an equal volume of cell culture medium (DMEM supplemented with 10% FBS, 20 mM glutamine, and penicillin/streptomycin). Cell suspensions were centrifuged, suspended with cell culture media, plated in T-75 flasks, and cultured at 37°C in 95% air and 5% CO_2_. Differentiation was induced after 1 day postconfluent cells (designated day 0) were treated with modified adipogenic differentiation medium containing 1 μM dexamethasone, 0.5 mM IBMX, 5 μg insulin/ml, and 0.5 μM rosiglitazone (Sigma Chemicals) for 48 h. After 48 h, maintenance medium containing 5 μg insulin/ml and 0.5 μM rosiglitazone nurtured the cells until day 6. MEFs isolated from Fst KO embryos were also treated with recombinant FST (0.5 μg/ml) (R&D Systems) and medium refreshed on alternate days. Early passage MEFs (*P* ≤ 3) were used for each experiment in order to avoid senescence.

### Genotyping

Genomic DNA was isolated from the heads of each harvested embryo using Direct Lysis reagent (Viagen Biotech, CA). Genotypes of embryos were confirmed by PCR using the following primer sets: *a*) 5′-ATCTATCGCCCTTGGGTCTT-3′ and 5′-AAAACCTACCGCAAC­­GAATG-3′, which amplifies a 152 bp fragment from the WT Fst allele; and *b*) 5′-GGTGGGAAATGTCACCTGAT-3′ and 5′-CGG­TG­GATGTGGAATGTGT-3′, which amplifies a 262 bp fragment from the Fst KO allele. The PCR cycle profile used for genotyping was as follows: 94°C for 1 min, 94°C for 30 s/68°C for 30 s (2 min for 15 cycles with −0.5°C per cycle), 94°C for 30 s, and 60°C for 30 s for 20 cycles (18).

### Real-time qPCR analysis

Quantitative gene expression analysis of mouse tissues and MEFs was performed by qPCR. Total RNA was extracted with Trizol reagent (Life Technologies, Carlsbad, CA) and equal amounts (2 μg) of RNA were reverse transcribed using a RNA high capacity cDNA kit (Applied Biosystems, Foster City, CA). The Power Sybr Green PCR master mix was used with a 7500 fast real-time PCR system (Applied Biosystems). The primer pairs were designed using Primer bank. Samples of 25 ng cDNA were analyzed in quadruplicate in parallel with GAPDH controls. The experimental mRNA starting quantities were calculated from standard curves and averaged using 7500 software v1.4 (18, 19).

### Oil Red O staining

Differentiated MEF cultures were washed in 1× PBS, fixed in 2% paraformaldehyde, and stained with 0.5% Oil Red O (Sigma Chemicals) for 15 min as described previously (24). Oil Red O staining was quantified by image analysis using the Image Pro 4.01 software (Media Cybernetics, Silver Spring, MD), coupled to a Leica DMLB microscope/VCC video camera. After images were calibrated for background lighting and relative Oil Red O optical density (OD) was calculated as integrated OD (IOD) (IOD = area × average intensity) using at least 20 pictures per treatment group (24).

### Immunoblot analysis

Proteins were resolved on 10–12% SDS-PAGE gels and then electrotransferred and analyzed for protein levels using the following antibodies: anti-C/EBP-α (1:1,000 dilution, catalog number sc-61; Santa Cruz Biotechnology, CA); anti-PPARγ (1:1,000 dilution, catalog number sc-7273; Santa Cruz Biotechnology), anti-Fst (1:5,000 dilution, a kind gift from Dr. Alan Schneyer, Pioneer Valley Life Science Institute, MA); anti-UCP1 (1:1,000 dilution, catalog number ab10983, Abcam, MA); anti-PRDM16 (1:300 dilution, catalog number sc-130243, Santa Cruz Biotechnology), anti-Cyt C (1:1,000 dilution, catalog number 42805; Cell Signaling, MA); anti-PPAR gamma coactivator-1α (PGC-1α) (1:1,000 dilution, catalog number sc-13067; Santa Cruz Biotechnology), anti-major urinary protein 1 (Mup1) (1:1,000 dilution, catalog number sc-66976; Santa Cruz Biotechnology); anti-aP2 [also known as fatty acid binding protein 4 (FABP4)] (1:500 dilution, catalog number sc-136150; Santa Cruz Biotechnology); anti-adiponectin (1:1,000 dilution, catalog number MAB3608; Millipore, Temecula, CA); anti-AMPK (1:1,000 dilution, catalog number 2532S; Cell Signaling), anti-pAMPK (1:1,000 dilution, catalog number 2531S; Cell Signaling), anti-β-actin (1:2,000 dilution, catalog number sc-130656; Santa Cruz Biotechnology), and anti-GAPDH (1:5,000 dilution, catalog number AB2302; Millipore). Appropriate HRP-linked secondary antibodies (1:1,000 dilution; Cell Signaling) were used and immuno-reactive bands were visualized, scanned, and analyzed by Image Quant software (18, 19).

### Immunohistochemical analysis

Embryo sections were fixed overnight in formalin, embedded in paraffin blocks, and sectioned. Tissue sections were stained with hematoxylin/eosin or with anti-UCP1 antibody (1:200 dilution, catalog number ab10983; Abcam) following standard procedures. Quantitative image analysis was performed by computerized densitometry using the ImagePro 4.01 program (Media Cybernetics) coupled to a Leica B microscope equipped with a Spot RT digital camera (Diagnostic Instruments, Portland, OR) (18, 19).

### Microarray analysis

Global gene expression microarray studies utilizing Affymetrix U133 Plus 2.0 array (Affymetrix, Santa Clara, CA) was performed in collaboration with the University of California at Los Angeles Clinical Microarray Core. MEFs from WT (Fst^+/+^) and Fst KO (Fst^−/−^) mice were differentiated under modified adipogenic conditions, and RNA isolated with TRIzol followed by Qiagen column purification (Qiagen, Valencia, CA). RNA integrity was evaluated by an Agilent 2100 bioanalyzer (Agilent Technologies, Palo Alto, CA) and purity and concentration were determined by NanoDrop 8000 (NanoDrop, Wilmington, DE). Subsequent data analyses were performed using the Partek Genomics Suite with the CEL files obtained from GeneChip® Console Software. The data were normalized using the robust multi-array average algorithm. Changes in gene expression (≥3.5-fold) for selected genes were confirmed by qPCR using the primer sequences reported previously (18, 19, 25, 26).

### Analysis of cellular oxygen consumption

Oxygen consumption rates (OCRs) of differentiated MEFs were measured in a Seahorse Bioscience XF24 extracellular flux analyzer (North Billerica, MA) (27, 28). Undifferentiated MEFs were seeded at 4 × 10^4^ cells per well in DMEM containing 10% FBS and incubated overnight at 37°C with 5% CO_2_. MEF adipogenic differentiation was performed for 8 days before measurement. The day of the assay, medium was replaced by unbuffered DMEM supplemented with 25 mM glucose and cells were incubated at 37°C in a CO_2_-free incubator for 2 h before the OCR was recorded. During the assay, 0.5 μM carbonyl cyanide-p-trifluoromethoxyphenylhydrazone (FCCP) was injected to deduce the maximal respiration capacity (27, 28). MEFs were treated with 10 nM CL 316,243 for 2 h prior to the OCR measurements in order to stimulate the brown-like adipocytes.

### Statistical analysis

Data are presented as mean ± SEM, and between group differences were analyzed by one-way ANOVA using GraphPad Prism version 5.3 or 6.0 (GraphPad Software, San Diego, CA). If the overall ANOVA revealed significant differences, then pair-wise comparisons between groups were performed by Newman-Keuls multiple group test. All comparisons were two-tailed and *P* ≤ 0.05 was considered statistically significant. The experiments were repeated at least three times (except for microarray analysis), and data from representative experiments are shown.

## RESULTS

### Analyses of Fst gene expression in various tissues

**Fig. 1.**
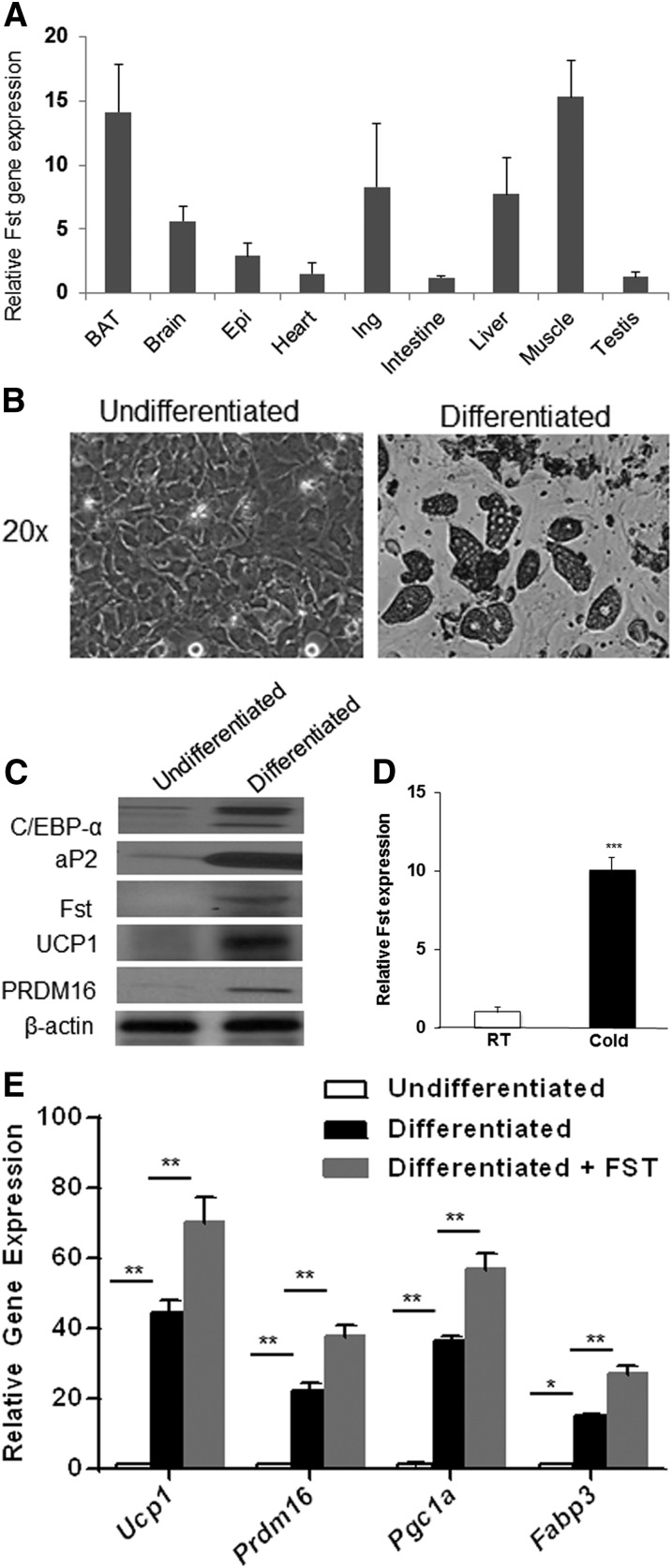
Tissue distribution of Fst and its induction during adipogenic differentiation of mouse brown preadipocyte cells. A: Real-time qPCR analysis of Fst gene expression in mouse tissue panel (n = 3). B: Photomicrographs of mouse brown preadipocyte cells grown either in regular growth medium (undifferentiated) or in BAT-specific adipogenic medium (differentiated) for 8 days. C: Analysis of key adipogenic markers in cell lysates obtained from undifferentiated and differentiated brown preadipocyte cells. Representative data from three independent experiments are shown. D: Fst mRNA levels in BAT from room temperature (RT) and 8 h cold-exposed (4–6°C) (Cold) mice (n = 3). E: Effect of recombinant FST protein (0.5 μg/ml) on selected genes in mouse brown preadipocyte cells during adipogenic differentiation (n = 3). Data are presented as mean ± SEM. *P ≤ 0.05; **P ≤ 0.01; ***P ≤ 0.001. Epi, epididymal fat; Ing, inguinal fat.

In order to gain some insight regarding possible functional roles of Fst in various tissue types, we performed real-time qPCR analysis of a tissue panel prepared from C57BL6/J mice. This panel contained BAT and epididymal and inguinal subcutaneous WAT depots, as well as several other metabolic tissues including brain, heart, intestine, liver, skeletal muscle, and testis. Fst gene expression was highest in BAT and skeletal muscle, and was also expressed at substantial levels in inguinal WAT and liver, with significant but lower levels in other tissues (**Fig. 1A**). While the role of Fst in regulating skeletal muscle mass is well-known, our data suggest a possible novel role for Fst in both BAT and WAT.

### Induction of Fst during BAT-specific differentiation of mouse brown preadipocyte cells and cold exposure

We investigated whether Fst expression is regulated during brown adipocyte differentiation. Mouse brown preadipocytes were allowed to differentiate for 8 days under brown adipocyte-specific conditions as previously described (23). As expected, the cells accumulated lipids in multilocular droplets (Fig. 1B), and levels of key brown adipocyte proteins (UCP1, PRDM16, C/EBP-α, and aP2) were significantly induced (Fig. 1C). Importantly, Fst protein levels increased from nearly undetectable in preadipocytes to substantial levels in differentiated brown adipocytes (Fig. 1C). Notably, Fst was also dramatically induced in BAT obtained from cold-exposed mice (Fig. 1D). Because Fst is a secreted protein, we tested whether exogenously added FST protein had an effect on brown adipocyte differentiation. Treatment of cells during differentiation with FST (0.5 μg/ml) enhanced thermogenic gene expression 50–80% beyond levels in cultures differentiated without FST (Fig. 1E). Thus, our data indicate a possible functional role of Fst during brown adipocyte differentiation and regulation of the thermogenic process.

### Impairment of both adipogenesis and induction of brown adipocyte markers during differentiation of Fst-deficient cells

**Fig. 2.**
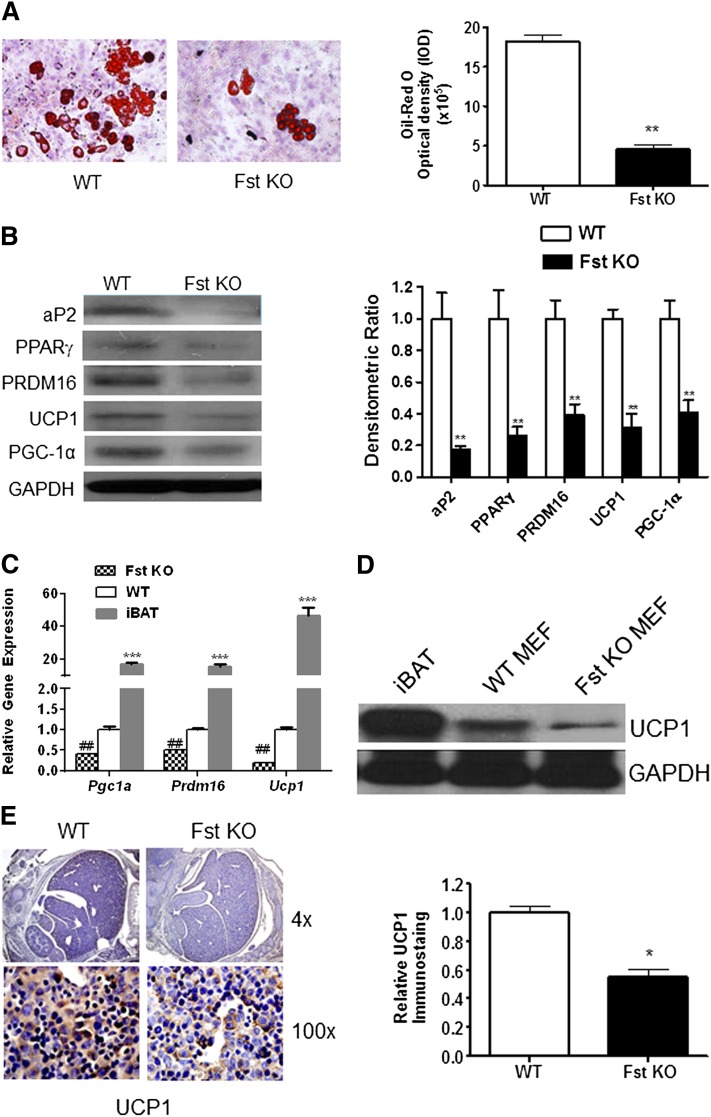
Inhibition of key thermogenic markers in differentiating Fst KO MEF cultures compared with the WT group. A: Left panel, Oil Red O staining. Right panel, quantitative image analysis showing relative Oil Red O OD expressed as IOD (IOD = total intensity × area of staining) of differentiated WT and Fst KO MEF cultures (n = 3). B: Left panel, Western blot analysis. Right panel, densitometric analysis of key adipogenic proteins expressed in WT and Fst KO MEF cultures undergoing adipogenic differentiation (n = 3). C: Real-time qPCR analysis comparing key thermogenic genes expressed in differentiated MEFs and interscapular BAT (iBAT) in vivo (n = 3). D: Comparison of UCP1 protein expression in differentiated WT and Fst KO MEFs and iBAT. Representative data from three independent experiments are shown. E: Left panel, immunohistochemical staining of UCP1 protein in WT and Fst KO embryo sections (day 14) is visible as brown colored regions. Right panel, quantitation of UCP1 staining density in stained sections as performed by computerized densitometry (see Materials and Methods for more detail) (n = 3). Data are presented as mean ± SEM. *P ≤ 0.05; **P ≤ 0.01; ***P ≤ 0.001; ##*P* ≤ 0.01 compared with WT group.

To assess whether Fst is required for the acquisition of brown adipocyte features during adipocyte differentiation, we compared adipogenesis in MEFs isolated from WT or Fst KO embryos. MEFs were used at early passage (≤3 passages), and independent isolates from distinct mothers gave similar results. MEFs were allowed to differentiate for 8 days using an established adipogenic cocktail (18). Compared with WT MEFs, Fst KO MEFs accumulated lower amounts of neutral lipids as detected by Oil Red O staining (**Fig. 2A**). Fst deficiency also led to lower levels of several key adipogenic proteins that are present in both WAT and BAT, including FABP4 (also known as aP2), PPARγ, PRDM16, UCP1, and PGC-1α (Fig. 2B). It should be noted that although clearly detectable levels of UCP1 and related proteins are induced upon WT MEF differentiation, these levels are far lower than those present in mouse interscapular BAT (Fig. 2C, D). Nevertheless, the MEF system allows assessment of the effect of Fst deficiency on adipogenic conversion, which is difficult to study in vivo due to perinatal death of Fst KO pups (22). Our results in MEF cells are further supported by the demonstration that e14 embryo sections from Fst KO mice exhibit approximately 50% lower levels of UCP1 immunostaining than WT embryos (Fig. 2E). Together, these results indicate that Fst influences adipogenesis and brown adipocyte differentiation.

### Recombinant Fst rescues thermogenic genes and protein levels in Fst KO MEFs

**Fig. 3.**
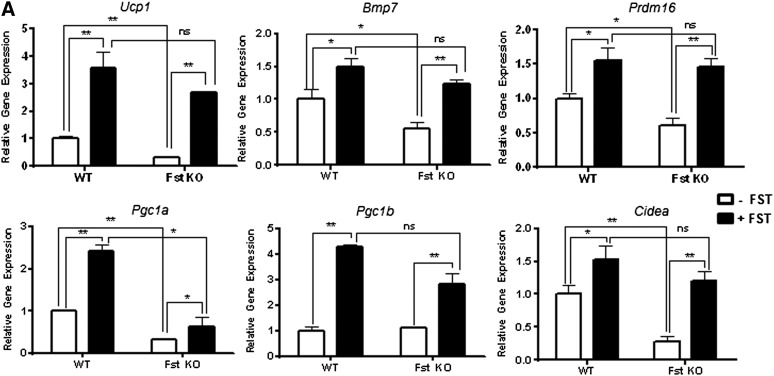

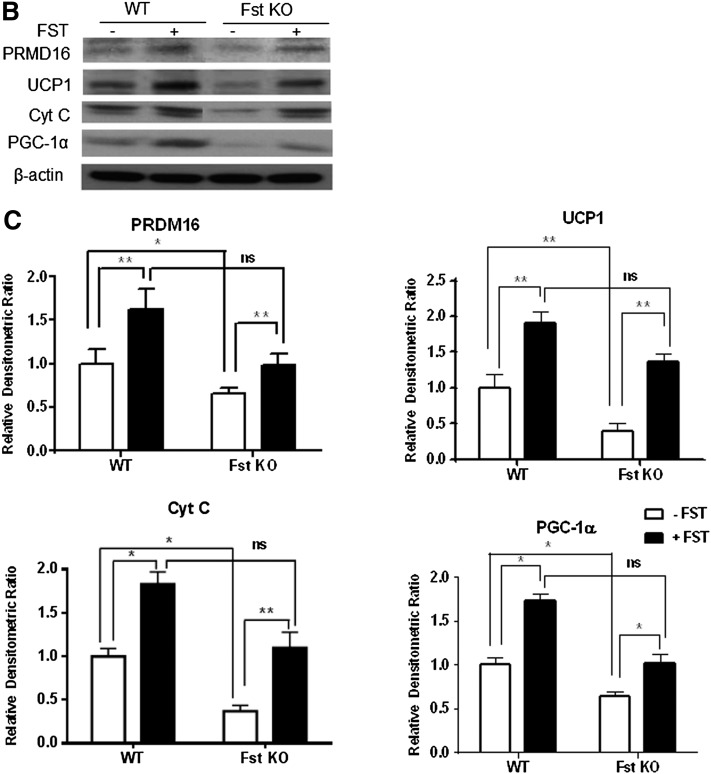
Analysis of key BAT-related genes and proteins in WT and Fst KO MEF cultures allowed to differentiate either in the presence or the absence of recombinant FST protein. A: Real-time qPCR analysis in WT and Fst KO MEF cultures after treatment with recombinant FST protein (0.5 μg/ml) after 4 days (n = 3). Western blot (B) and densitometric analysis (C) of PRDM16, UCP1, Cyt C, and PGC-1α proteins in differentiating MEF cultures from WT and Fst KO groups (n = 3). Data are presented as mean ± SEM. *P ≤ 0.05; **P ≤ 0.01. ns, nonsignificant.

To confirm that the difference in adipogenic capacity between Fst KO and WT MEFs was attributable to lack of Fst, we treated WT and Fst KO MEFs with FST (0.5 μg/ml) at the beginning of differentiation for 4 days. FST treatment led to significant increases in adipocyte gene expression and protein levels over differentiation medium alone (**Fig. 3**). Importantly, the addition of FST to Fst KO cultures restored or exceeded the mRNA levels of several brown adipocyte markers including *Ucp1*, *Bmp7*, *Prdm16*, *Pgc1a*, *Pgc1b*, and *Cidea* (Fig. 3A) and protein levels of PRDM16, UCP1, Cyt-C, and PGC-1α observed in WT MEFs treated with adipogenic medium (Fig. 3B, C). Interestingly, WT MEFs also exhibited enhanced mRNA and protein levels in response to FST. There was no significant difference in gene expression after FST treatment between WT and Fst KO groups except for *Pgc1a* (Fig. 3A). These results are in agreement with our finding in the brown adipocyte cell line (Fig. 1).

### Global gene expression profiling demonstrates significant lack of stimulation of key genes involved in lipid and energy metabolism in Fst KO MEFs compared with the WT group

**Fig. 4.**
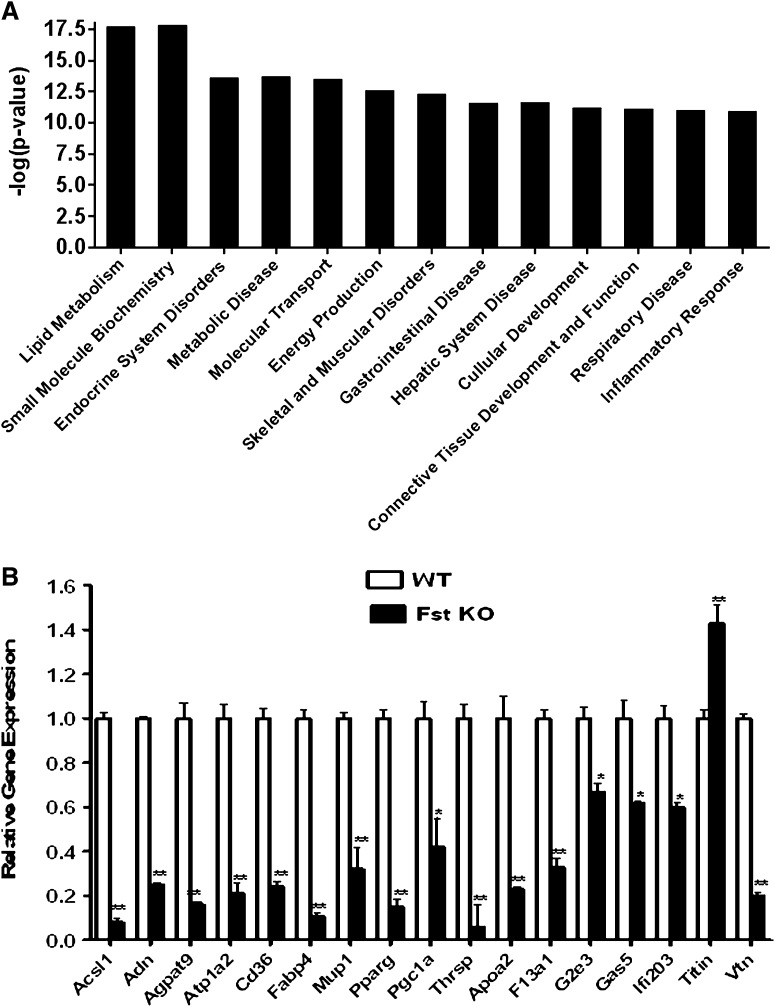
A: Ingenuity pathway analysis demonstrating lipid metabolism as the most significantly altered pathway between WT versus Fst KO groups (n = 1). B: Validation of Affymetrix gene expression analysis by qPCR using gene-specific primers (n = 3). Data are presented as mean ± SEM. *P ≤ 0.05; **P ≤ 0.01.

Based on our initial findings that Fst loss-of-function impairs induction of key BAT-related genes and proteins, we sought to more broadly determine the effects of Fst on gene expression during early stages of brown adipocyte induction. We performed Affymetrix global gene expression profiling of WT and Fst KO MEFs after treatment with differentiation medium for 48 h. An unbiased analysis of the pathways affected by Fst using Ingenuity Pathway Analysis revealed most prominent differences between the two genotypes in mRNA levels for genes implicated in lipid metabolism and energy metabolism pathways (**Fig. 4A**). Expression levels for genes having a reduction of 3.5-fold or more in Fst KO compared with WT MEFs (**Table 1**) were measured by qPCR. We confirmed significant downregulation of several genes including *Acsl1*, *Adn* (adiponectin), *Agpat9*, *Atpa2*, *Cd36*, *Fabp4*, *Mup1*, *Pparg*, *Thrsp*, *Apoa2*, and *Pgc1a* (Fig. 4B). All additional differentially expressed genes involved in lipid and energy metabolism with changes less than 3.5-fold but higher than 1.5-fold in magnitude are shown in supplementary Table I.

**TABLE 1.** Downregulation of energy production- and lipid metabolism-related genes in differentiating Fst KO MEF cultures compared with the WT group

Symbol	Entrez Gene Name	Fst KO versus Fst WT (fold change)
*Adn*	Adiponectin, C1Q and collagen domain containing	−36.7
*Thrsp*	Thyroid hormone responsive	−11.7
*Hp*	Haptoglobin	−10.1
*Acsl1*	Acyl-CoA synthetase long-chain family member 1	−9.0
*Fabp4*	Fatty acid binding protein 4, adipocyte	−6.3
*Agpat9*	1-Acylglycerol-3-phosphate O-acyltransferase 9	−4.8
*Plg*	Plasminogen	−4.8
*Pparg*	PPARγ	−4.7
*Apoc2*	Apolipoprotein C-II	−4.2
*Atp1a2*	ATPase, Na^+^/K^+^ transporting, α2 polypeptide	−4.2
*Saa1*	Serum amyloid A1	−4.1
*Cps1*	Carbamoyl-phosphate synthase 1, mitochondrial	−4.0
*Cd36*	CD36 molecule (thrombospondin receptor)	−3.9
*Mup1*	Major urinary protein 1	−3.9
*Serpina1*	Serpin peptidase inhibitor clade A (α-1 antiproteinase, antitrypsin) member 1	−3.6

Cells were allowed to differentiate under BAT-specific conditions as described in the Material and Methods and Affymetrix gene expression analysis was performed. Changes in energy production- and lipid metabolism-specific genes that were above 3.5-fold were selected.

### Decreased respiration in differentiating Fst-deficient MEFs is enhanced by restoration of Fst

**Fig. 5.**
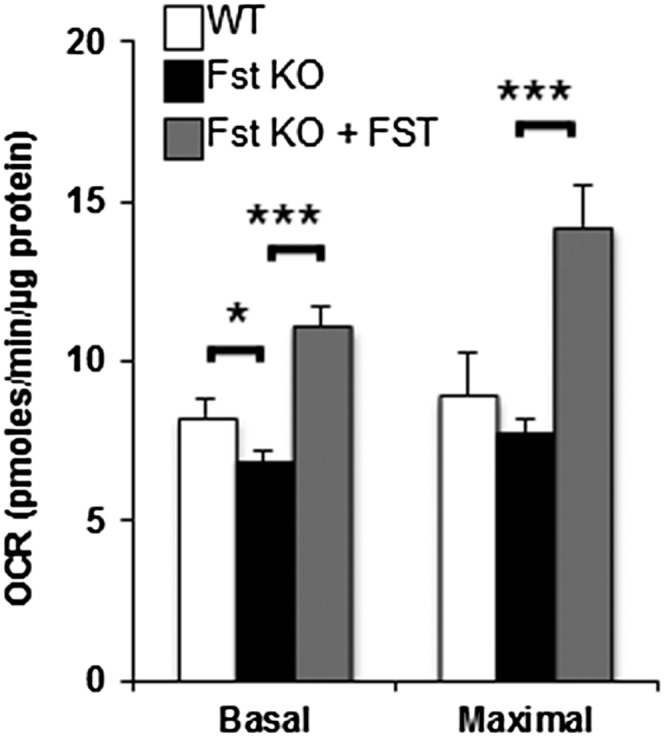
Measurements of OCR in differentiated WT and Fst KO MEFs. Eight day-differentiated MEFs were activated with 10 nM CL 316,243 for 2 h prior to OCR measurements. Basal respiration and maximal respiration capacity were obtained before and after FCCP injection, respectively (n = 3). Data are expressed as mean ± SEM. *P ≤ 0.05; ***P ≤ 0.001.

We evaluated OCR in differentiating WT and Fst KO MEFs to determine whether the alterations in gene and protein levels observed in Fst deficiency are reflected in cellular respiration and to further test whether recombinant FST could restore cellular respiration in Fst-deficient MEFs. WT and Fst KO MEFs were differentiated for 8 days and cellular respiration was measured with an XF24 analyzer. OCR was recorded under both basal and FCCP-treated conditions in order to obtain the maximal respiration capacity. As shown in **Fig. 5**, Fst KO cells exhibited significantly lower basal mitochondrial respiration compared with the WT cells. Importantly, the addition of recombinant FST to Fst KO cultures rescued the respiration impairment, allowing the OCR to even exceed the levels of WT cells. The recombinant FST also increased maximal OCR in Fst KO cells. These results indicate that Fst increases cellular respiration.

## DISCUSSION

Fst is an extracellular protein that binds activins and Mst with high affinity and inhibits signaling through TGF-β superfamily members that regulate diverse processes such as cell growth and differentiation, as well as secretion of follicle stimulating hormone (29–31). Fst KO mice die shortly after birth (22), and therefore have been difficult to study. Although the role of Fst in promoting muscle mass is well-documented (19, 32–34), its role in other metabolic tissues is less well-known. We identified Fst expression in BAT and WAT, and investigated its potential role in adipocyte biology. Our studies revealed for the first time that Fst is induced upon differentiation of brown preadipocytes, and that FST protein treatment during adipocyte differentiation increased mRNA and protein levels of thermogenic genes that are characteristic of brown and beige adipocytes. Moreover, Fst expression was substantially induced in BAT from cold-exposed mice. More importantly, these results may have broader implications for a role of Fst in brown adipocyte biology.

Recent studies convincingly demonstrate that inhibition of Mst, and related members of the TGF-β superfamily, promotes browning of WAT and protects mice from diet-induced obesity and insulin resistance (14, 18, 20, 21). Because Fst is a known antagonist of activins and Mst, we investigated the role of Fst during BAT differentiation and browning of WAT using appropriate model systems. While classical BAT develops from Myf5-positive precursors that differentiate into brown adipocytes through the action of PRDM16, beige adipocytes responsible for browning of WAT arise from non-Myf5 cell lineage (35–38). These beige adipocytes have characteristics of both white and brown fat cells with a unique gene expression profile and cell surface proteins (38). A systematic approach employed in this study, by initially analyzing the expression of Fst in mouse tissue panel and subsequently in differentiating mouse brown preadipocyte cells and MEF primary cultures, demonstrated a novel role for Fst in regulating brown fat activity. We utilized a modified adipogenic differentiation cocktail medium that contained rosiglitazone along with regular adipogenic cocktail that included IBMX, insulin, and dexamethasone to induce brown fat-like differentiation of MEFs (18). Rosiglitazone, a PPARγ agonist, is known to induce mitochondrial biogenesis (39), white-to-brown fat conversion (15), and nonadrenergic recruitment of brown preadipocytes (40, 41). Using this modified adipogenic differentiation cocktail, we found brown fat-like multilocular adipocytes that expressed BAT-specific gene signature and also expressed key markers involved in lipid and energy metabolism. We showed that Fst loss-of-function in MEF cultures significantly decreased the expression of adipogenic markers and key thermogenic markers, the latter being rescued by simultaneous treatment of the differentiating cells with recombinant FST. These results support the role of Fst in the acquisition of BAT-like phenotypes. Downregulation of several key genes involved in lipid and energy metabolism in the Fst KO groups suggests an important role of Fst during this process. Fst KO MEFs also had significantly lower levels of phosphorylated adenosine monophosphate-activated protein kinase (pAMPK) (supplementary Fig. I), which acts as a metabolic fuel gauge and plays an important role in regulating cellular energy balance (42). Therefore, our global gene profiling data indicates that Fst is a key modulator of lipid and energy metabolism and its loss-of-function results in severe metabolic defects that may be essential for survival.

The molecular mechanisms responsible for Fst-induced brown characteristics of white adipocytes are not known at the moment; however, it is possible that Fst may antagonize the Mst/TGF-β signaling pathway during the process. Mst is predominantly expressed in skeletal muscle and plays an important role in regulation of muscle mass and fat deposition (43). We have recently reported that Mst inactivation in Mst KO MEFs promotes pathways related to lipid metabolism and brown fat differentiation (18). It will be interesting to explore whether Fst targets Mst signaling to promote adipocyte differentiation and browning. Among several relevant genes involved in regulating brown characteristics, we also identified PGC-1α and Mup1 as two interesting genes with established roles during regulation of energy homeostasis. While PGC-1α is known to drive brown fat-like development of white fat and influence thermogenesis (44, 45), Mup1, a member of the lipocalin family, has recently been identified as a key regulator of glucose and lipid metabolism and increases energy expenditure through enhanced mitochondrial functions (46–48). In spite of the limitations of using MEFs as our model systems, our data clearly identified a novel role of Fst in regulating both protein and gene expression of these established regulators of thermogenic programs. A growing list of agents have recently been reported to promote browning of WAT, a process that engages several distinct mechanisms to regulate white adipose function, energy expenditure, and regulation of certain aspects of thermogenic programs that are linked to their ability to resist diet-induced obesity and improved insulin sensitivity (13, 16, 49). Fst is known to promote skeletal muscle mass, which is abundant in Myf5-expressing cells of myogenic lineage. Although it is not known whether Fst regulates the Myf5-positive precursor pool present in skeletal muscle and classical brown fat, it is possible that it may also promote classical BAT mass and its activity by directly targeting Myf5-expressing cells.

Although our in vitro data obtained from differentiated brown adipocyte and MEF cultures suggest significant induction of genes and proteins involved in maintaining energy homeostasis and increased cellular respiration in the presence of Fst, detailed studies examining key metabolic parameters in Fst transgenic mice would be extremely important to ascertain its functional role in vivo. It is important to generate Fst transgenic mice under both white adipocyte-specific adiponectin promoters as well as under brown adipocyte-specific UCP1-specific promoters in order to identify the potential adipocyte target of Fst. Such in vivo models would clearly delineate potential tissue targets of Fst, as well as its precise mechanism of action and its effect on various metabolic parameters including whole body energy expenditure, glucose clearance, and insulin sensitivity, as well as the effect of high-fat diet on the body composition during its regulation of energy homeostasis. Understanding the molecular targets responsible for Fst’s action during its regulation of energy homeostasis may provide a novel therapeutic approach to treat obesity and type 2 diabetes.

## Supplementary Material

Supplemental Data

## References

[1] Tseng Y-H. Cypess A. M. Kahn C. R. 2010 Cellular bioenergetics as a target for obesity therapy. Nat. Rev. Drug Discov. 9: 465–482 10.1038/nrd3138 PMC2880836 20514071

[2] Haslam D. W. James W. P. 2005 Obesity. Lancet. 366: 1197–1209 10.1016/S0140-6736(05)67483-1 16198769

[3] Bhatia L. S. Curzen N. P. Byrne C. D. 2012 Nonalcoholic fatty liver disease and vascular risk. Curr. Opin. Cardiol. 27: 420–428 22596186 10.1097/HCO.0b013e328354829c

[4] Isidro M. L. Cordido F. 2009 Drug treatment of obesity: established and emerging therapies. Mini Rev. Med. Chem. 9: 664–673 10.2174/138955709788452739 19519492

[5] Grundy S. M. 2006 Drug therapy of the metabolic syndrome: minimizing the emerging crisis in polypharmacy. Nat. Rev. Drug Discov. 5: 295–309 10.1038/nrd2005 16582875

[6] Whittle A. J. López M. Vidal-Puig A. 2011 Using brown adipose tissue to treat obesity - the central issue. Trends Mol. Med. 17: 405–411 10.1016/j.molmed.2011.04.001 21602104

[7] Boss O. Farmer S. R. 2012 Recruitment of brown adipose tissue as a therapy for obesity-associated diseases. Front. Endocrinol. (Lausanne). 3: 14 10.3389/fendo.2012.00014 PMC3356088 22654854

[8] Kajimura S. Seale P. Spiegelman B. M. 2010 Transcriptional control of brown fat development. Cell Metab. 11: 257–262 10.1016/j.cmet.2010.03.005 PMC2857670 20374957

[9] Nedergaard J. Bengtsson T. Cannon B. 2011 New powers of brown fat: fighting the metabolic syndrome. Cell Metab. 13: 238–240 10.1016/j.cmet.2011.02.009 21356513

[10] Bartelt A. Bruns O. T. Reimer R. Hohenberg H. Ittrich H. Peldschus K. Kaul M. G. Tromsdorf U. I. Weller H. Waurisch C. 2011 Brown adipose tissue activity controls triglyceride clearance. Nat. Med. 17: 200–205 10.1038/nm.2297 21258337

[11] Hansen J. B. Kristiansen K. 2006 Regulatory circuits controlling white versus brown adipocyte differentiation. Biochem. J. 398: 153–168 16898874 10.1042/BJ20060402 PMC1550312

[12] Frontini A. Cinti S. 2010 Distribution and development of brown adipocytes in the murine and human adipose organ. Cell Metab. 11: 253–256 10.1016/j.cmet.2010.03.004 20374956

[13] Ohno H. Shinoda K. Spiegelman B. M. Kajimura S. 2012 PPARγ agonists induce a white-to-brown fat conversion through stabilization of PRDM16 protein. Cell Metab. 15: 395–404 10.1016/j.cmet.2012.01.019 PMC3410936 22405074

[14] Shan T. Liang X. Bi P. Kuang S. 2013 Myostatin knockout drives browning of white adipose tissue through activating the AMPK-PGC1α-Fndc5 pathway in muscle. FASEB J. 27: 1981–1989 10.1096/fj.12-225755 PMC3633817 23362117

[15] Gburcik V. Cleasby M. E. Timmons J. A. 2013 Loss of neuronatin promotes “browning” of primary mouse adipocytes while reducing Glut1-mediated glucose disposal. Am. J. Physiol. Endocrinol. Metab. 304: E885–E894 10.1152/ajpendo.00463.2012 PMC3625784 23482445

[16] Cao L. Choi E. Y. Liu X. Martin A. Wang C. Xu X. During M. J. 2011 White to brown fat phenotypic switch induced by genetic and environmental activation of a hypothalamic-adipocyte axis. Cell Metab. 14: 324–338 10.1016/j.cmet.2011.06.020 PMC3172615 21907139

[17] Qiang L. Wang L. Kon N. Zhao W. Lee S. Zhang Y. Rosenbaum M. Zhao Y. Gu W. Farmer S. R. 2012 Brown remodeling of white adipose tissue by SirT1-dependent deacetylation of Pparγ. Cell. 150: 620–632 10.1016/j.cell.2012.06.027 PMC3413172 22863012

[18] Braga M. Pervin S. Norris K. Bhasin S. Singh R. 2013 Inhibition of in vitro and in vivo brown fat differentiation program by myostatin. Obesity (Silver Spring). 21: 1180–1188 10.1002/oby.20117 PMC3735638 23868854

[19] Singh R. Bhasin S. Braga M. Artaza J. N. Pervin S. Taylor W. E. Krishnan V. Sinha S. K. Rajavashisth T. B. Jasuja R. 2009 Regulation of myogenic differentiation by androgens: cross-talk between androgen receptor/ beta-catenin and follistatin/transforming growth factor-beta signaling pathways. Endocrinology. 150: 1259–1268 10.1210/en.2008-0858 PMC2654730 18948405

[20] Yadav H. Quijano C. Kamaraju A. K. Gavrilova O. Malek R. Chen W. Zerfas P. Zhigang D. Wright E. C. Stuelten C. 2011 Protection from obesity and diabetes by blockade of TGF-β/Smad3 signaling. Cell Metab. 14: 67–79 10.1016/j.cmet.2011.04.013 PMC3169298 21723505

[21] Zhang C. McFarlane C. Lokireddy S. Masuda S. Ge X. Gluckman P. D. Sharma M. Kambadur R. 2012 Inhibition of myostatin protects against diet-induced obesity by enhancing fatty acid oxidation and promoting a brown adipose phenotype in mice. Diabetologia. 55: 183–193 10.1007/s00125-011-2304-4 21927895

[22] Matzuk M. M. Lu N. Vogel H. Sellheyer K. Roop D. R. Bradley A. 1995 Multiple defects and perinatal death in mice deficient in follistatin. Nature. 374: 360–363 10.1038/374360a0 7885475

[23] Uldry M. Yang W. St-Pierre J. Lin J. Seale P. Spiegelman B. M. 2006 Complementary action of the PGC-1 coactivators in mitochondrial biogenesis and brown fat differentiation. Cell Metab. 3: 333–341 10.1016/j.cmet.2006.04.002 16679291

[24] Singh R. Artaza J. N. Taylor W. E. Braga M. Yuan X. Gonzalez-Cadavid N. F. Bhasin S. 2006 Testosterone inhibits adipogenic differentiation in 3T3-L1 cells: nuclear translocation of androgen receptor complex with beta-catenin and T-cell factor 4 may bypass canonical Wnt signaling to down-regulate adipogenic transcription factors. Endocrinology. 147: 141–154 10.1210/en.2004-1649 PMC4417624 16210377

[25] Koh S. S. Wei J. P. Li X. Huang R. R. Doan N. B. Scolyer R. A. Cochran A. J. Binder S. W. 2012 Differential gene expression profiling of primary cutaneous melanoma and sentinel lymph node metastases. Mod. Pathol. 25: 828–837 10.1038/modpathol.2012.32 22411186

[26] Donahue T. R. Tran L. M. Hill R. Li Y. Kovochich A. Calvopina J. H. Patel S. G. Wu N. Hindoyan A. Farrell J. J. 2012 Integrative survival-based molecular profiling of human pancreatic cancer. Clin. Cancer Res. 18: 1352–1363 10.1158/1078-0432.CCR-11-1539 PMC3816537 22261810

[27] Plaisier C. L. Bennett B. J. He A. Guan B. Lusis A. J. Reue K. Vergnes L. 2012 Zbtb16 has a role in brown adipocyte bioenergetics. Nutr. Diabetes. 2: e46 10.1038/nutd.2012.21 PMC3461357 23446662

[28] Kidani Y. Elsaesser H. Hock M. B. Vergnes L. Williams K. J. Argus J. P. Marbois B. N. Komisopoulou E. Wilson E. B. Osborne T. F. 2013 Sterol regulatory element-binding proteins are essential for the metabolic programming of effector T cells and adaptive immunity. Nat. Immunol. 14: 489–499 10.1038/ni.2570 PMC3652626 23563690

[29] Lee S. J. Lee Y. S. Zimmers T. A. Soleimani A. Matzuk M. M. Tsuchida K. Cohn R. D. Barton E. R. 2010 Regulation of muscle mass by follistatin and activins. Mol. Endocrinol. 24: 1998–2008 10.1210/me.2010-0127 PMC2954636 20810712

[30] Schneyer A. L. Sidis Y. Gulati A. Sun J. L. Keutmann H. Krasney P. A. 2008 Differential antagonism of activin, myostatin and growth and differentiation factor 11 by wild-type and mutant follistatin. Endocrinology. 149: 4589–4595 18535106 10.1210/en.2008-0259 PMC2553374

[31] Ying S. Y. 1988 Inhibins, activins, and follistatins: gonadal proteins modulating the secretion of follicle-stimulating hormone. Endocr. Rev. 9: 267–293 10.1210/edrv-9-2-267 3136011

[32] Braga M. Bhasin S. Jasuja R. Pervin S. Singh R. 2012 Testosterone inhibits transforming growth factor-β signaling during myogenic differentiation and proliferation of mouse satellite cells: potential role of follistatin in mediating testosterone action. Mol. Cell. Endocrinol. 350: 39–52 10.1016/j.mce.2011.11.019 PMC3264813 22138414

[33] Winbanks C. E. Weeks K. L. Thomson R. E. Sepulveda P. V. Beyer C. Qian H. Chen J. L. Allen J. M. Lancaster G. I. Febbraio M. A. 2012 Follistatin-mediated skeletal muscle hypertrophy is regulated by Smad3 and mTOR independently of myostatin. J. Cell Biol. 197: 997–1008 10.1083/jcb.201109091 PMC3384410 22711699

[34] Gilson H. Schakman O. Kalista S. Lause P. Tsuchida K. Thissen J. P. 2009 Follistatin induces muscle hypertrophy through satellite cell proliferation and inhibition of both myostatin and activin. Am. J. Physiol. Endocrinol. Metab. 297: E157–E164 19435857 10.1152/ajpendo.00193.2009

[35] Cannon B. Nedergaard J. 2004 Brown adipose tissue: function and physiological significance. Physiol. Rev. 84: 277–359 14715917 10.1152/physrev.00015.2003

[36] Seale P. Bjork B. Yang W. Kajimura S. Chin S. Kuang S. Scimè A. Devarakonda S. Conroe H. M. Erdjument-Bromage H. 2008 PRDM16 controls a brown fat/skeletal muscle switch. Nature. 454: 961–967 10.1038/nature07182 PMC2583329 18719582

[37] Timmons J. A. Wennmalm K. Larsson O. Walden T. B. Lassmann T. Petrovic N. Hamilton D. L. Gimeno R. E. Wahlestedt C. Baar K. 2007 Myogenic gene expression signature establishes that brown and white adipocytes originate from distinct cell lineages. Proc. Natl. Acad. Sci. USA. 104: 4401–4406 10.1073/pnas.0610615104 PMC1810328 17360536

[38] Wu J. Boström P. Sparks L. M. Ye L. Choi J. H. Giang A. H. Khandekar M. Virtanen K. A. Nuutila P. Schaart G. 2012 Beige adipocytes are a distinct type of thermogenic fat cell in mouse and human. Cell. 150: 366–376 10.1016/j.cell.2012.05.016 PMC3402601 22796012

[39] Pardo R. Enguix N. Lasheras J. Feliu J. E. Kralli A. Villena J. A. 2011 Rosiglitazone-induced mitochondrial biogenesis in white adipose tissue is independent of peroxisome proliferator-activated receptor γ coactivator-1α. PLoS ONE. 6: e26989 10.1371/journal.pone.0026989 PMC3210129 22087241

[40] Petrovic N. Shabalina I. G. Timmons J. A. Cannon B. Nedergaard J. 2008 Thermogenically competent nonadrenergic recruitment in brown preadipocytes by a PPARgamma agonist. Am. J. Physiol. Endocrinol. Metab. 295: E287–E296 10.1152/ajpendo.00035.2008 18492776

[41] Schulz T. J. Huang T. L. Tran T. T. Zhang H. Townsend K. L. Shadrach J. L. Cerletti M. McDougall L. E. Giorgadze N. Tchkonia T. 2011 Identification of inducible brown adipocyte progenitors residing in skeletal muscle and white fat. Proc. Natl. Acad. Sci. USA. 108: 143–148 21173238 10.1073/pnas.1010929108 PMC3017184

[42] Cantó C. Gerhart-Hines Z. Feige J. N. Lagouge M. Noriega L. Milne J. C. Elliott P. J. Puigserver P. Auwerx J. 2009 AMPK regulates energy expenditure by modulating NAD+ metabolism and SIRT1 activity. Nature. 458: 1056–1060 19262508 10.1038/nature07813 PMC3616311

[43] Lee S. J. McPherron A. C. 2001 Regulation of myostatin activity and muscle growth. Proc. Natl. Acad. Sci. USA. 98: 9306–9311 11459935 10.1073/pnas.151270098 PMC55416

[44] Boström P. Wu J. Jedrychowski M. P. Korde A. Ye L. Lo J. C. Rasbach K. A. Boström E. A. Choi J. H. Long J. Z. 2012 A PGC1-α-dependent myokine that drives brown-fat-like development of white fat and thermogenesis. Nature. 481: 463–468 10.1038/nature10777 PMC3522098 22237023

[45] Liang H. Ward W. F. 2006 PGC-1alpha: a key regulator of energy metabolism. Adv. Physiol. Educ. 30: 145–151 17108241 10.1152/advan.00052.2006

[46] Hui X. Zhu W. Wang Y. Lam K. S. Zhang J. Wu D. Kraegen E. W. Li Y. Xu A. 2009 Major urinary protein-1 increases energy expenditure and improves glucose intolerance through enhancing mitochondrial function in skeletal muscle of diabetic mice. J. Biol. Chem. 284: 14050–14057 10.1074/jbc.M109.001107 PMC2682853 19336396

[47] Zhou Y. Jiang L. Rui L. 2009 Identification of MUP1 as a regulator for glucose and lipid metabolism in mice. J. Biol. Chem. 284: 11152–11159 10.1074/jbc.M900754200 PMC2670120 19258313

[48] Knopf J. L. Gallagher J. F. Held W. A. 1983 Differential, multihormonal regulation of the mouse major urinary protein gene family in the liver. Mol. Cell. Biol. 3: 2232–2240 10.1128/mcb.3.12.2232-2240.1983 PMC370094 6656765

[49] Bordicchia M. Liu D. Amri E. Z. Ailhaud G. Dessì-Fulgheri P. Zhang C. Takahashi N. Sarzani R. Collins S. 2012 Cardiac natriuretic peptides act via p38 MAPK to induce the brown fat thermogenic program in mouse and human adipocytes. J. Clin. Invest. 122: 1022–1036 10.1172/JCI59701 PMC3287224 22307324

