# Supplementary material for: Follistatin promotes adipocyte differentiation, browning, and energy metabolism
Source: J Lipid Res. 2014 Mar;55(3):375–84. doi: 10.1194/jlr.M039719 (PMC3934723; doi:10.1194/jlr.M039719)
Supplement: Supplemental Data [file supp_55_3_375__index.html]

Follistatin promotes adipocyte differentiation, browning, and energy metabolism — Follistatin promotes adipocyte differentiation, browning, and energy metabolism — Supplemental Data 

# Follistatin promotes adipocyte differentiation, browning, and energy metabolism

## Supplemental Data

**Files in this Data Supplement:**

- Supplemental Table 1 - Supplemental Table 1 List of differentially expressed genes in Fst KO MEFs
- Supplemental Figure 1 - Western blot analysis
