# Supplementary material for: Follistatin promotes adipocyte differentiation, browning, and energy metabolism
Source: J Lipid Res. 2014 Mar;55(3):375–84. doi: 10.1194/jlr.M039719 (PMC3934723; doi:10.1194/jlr.M039719)
Supplement: Supplemental Data [file supp_M039719_jlr.M039719-1.pdf]

Supplement Table 1: Down-regulation of energy production and lipid metabolism genes in Fst KO MEFs

Symbol	Entrez Gene Name	Fst KO vs WT (Fold Change)
A130040M12Rik	RIKEN cDNA A130040M12 gene	-1.861
ABCB1	ATP-binding cassette, sub-family B (MDR/TAP), member 1	-1.588
ABCD2	ATP-binding cassette, sub-family D (ALD), member 2	-1.916
ABHD5	abhydrolase domain containing 5	-1.556
ACE	angiotensin I converting enzyme (peptidyl-dipeptidase A) 1	-1.697
ACLY	ATP citrate lyase	1.529
ADH7	alcohol dehydrogenase 7 (class IV), mu or sigma polypeptide	-1.876
ADH1C	alcohol dehydrogenase 1C (class I), gamma polypeptide	-1.807
AFP	alpha-fetoprotein	-2.829
AGPAT2	1-acylglycerol-3-phosphate O-acyltransferase 2 (lysophosphatidic acid acyltransferase, beta)	-2.327
AGT	angiotensinogen (serpin peptidase inhibitor, clade A, member 8)	-2.597
ALDOB	aldolase B, fructose-bisphosphate	-1.634
ALB	albumin	-2.854
AMBP	alpha-1-microglobulin/bikunin precursor	-2.955
ANG	angiogenin, ribonuclease, RNase A family, 5	3.004
ANGPTL4	angiopoietin-like 4	-2.405
APOA1	apolipoprotein A-I	-3.189
APOA2	apolipoprotein A-II	-2.810
APOC1	apolipoprotein C-I	-3.153
APOD	apolipoprotein D	-1.715
APOE	apolipoprotein E	-1.671
APOH	apolipoprotein H (beta-2-glycoprotein I)	-1.971
ASPG	asparaginase homolog (S. cerevisiae)	-1.682
C3	complement component 3	-3.743
CAV2	caveolin 2	-1.584
CCL2	chemokine (C-C motif) ligand 2	-1.503
CCL11	chemokine (C-C motif) ligand 11	-2.725
CD7	CD7 molecule	1.540
CD14	CD14 molecule	-1.535
CEBPA	CCAAT/enhancer binding protein (C/EBP), alpha	-2.151
CIDEA	cell death-inducing DFFA-like effector a	-2.615
CIDEC	cell death-inducing DFFA-like effector c	-2.418
CLEC11A	C-type lectin domain family 11, member A	1.697
CP	ceruloplasmin (ferroxidase)	-2.269
CSF1R	colony stimulating factor 1 receptor	-1.520
CYB5A	cytochrome b5 type A (microsomal)	-1.601
CYP11B1	cytochrome P450, family 11, subfamily B, polypeptide 1	-1.621
CYP2F1	cytochrome P450, family 2, subfamily F, polypeptide 1	-1.545
CYP2J2	cytochrome P450, family 2, subfamily J, polypeptide 2	-1.563
CYP3A4	cytochrome P450, family 3, subfamily A, polypeptide 4	-3.377
CYP3A43	cytochrome P450, family 3, subfamily A, polypeptide 43	-2.888
CYP7B1	cytochrome P450, family 7, subfamily B, polypeptide 1	-1.798
DGAT2	diacylglycerol O-acyltransferase 2	-2.645
DKK1	dickkopf homolog 1 (Xenopus laevis)	1.541
DRD1	dopamine receptor D1	-1.597
DSP	desmoplakin	1.519
EDNRB	endothelin receptor type B	-1.791

Supplement Table 1: Down-regulation of energy production and lipid metabolism genes in Fst KO MEFs

Symbol	Entrez Gene Name	Fst KO vs WT (Fold Change)
ELOVL2	ELOVL fatty acid elongase 2	-2.693
ENPP2	ectonucleotide pyrophosphatase/phosphodiesterase 2	-1.549
EPAS1	endothelial PAS domain protein 1	-1.615
EPHX2	epoxide hydrolase 2, cytoplasmic	-1.739
F2	coagulation factor II (thrombin)	-1.822
FABP1	fatty acid binding protein 1, liver	-2.549
FCGR2A	Fc fragment of IgG, low affinity IIa, receptor (CD32)	-1.642
Fcrls	Fc receptor-like S, scavenger receptor	1.506
FGF9	fibroblast growth factor 9 (glia-activating factor)	1.603
FOXO4	forkhead box O4	1.565
FMO1	flavin containing monooxygenase 1	-1.581
FMO2	flavin containing monooxygenase 2 (non-functional)	-1.635
G6PC	glucose-6-phosphatase, catalytic subunit	-1.576
GFRA2	GDNF family receptor alpha 2	-1.793
GPD1	glycerol-3-phosphate dehydrogenase 1 (soluble)	-3.453
HPGD	hydroxyprostaglandin dehydrogenase 15-(NAD)	-1.740
HPX	hemopexin	-2.628
HSD3B2	hydroxy-delta-5-steroid dehydrogenase, 3 beta- and steroid delta-isomerase 2	-1.693
IL1R1	interleukin 1 receptor, type I	-1.629
KITLG	KIT ligand	-2.049
KLF2	Kruppel-like factor 2 (lung)	1.518
LECT1	leukocyte cell derived chemotaxin 1	-3.351
LIPE	lipase, hormone-sensitive	-2.595
LIPH	lipase, member H	-1.642
LPL	lipoprotein lipase	-2.548
MAOB	monoamine oxidase B	-1.517
MGST2	microsomal glutathione S-transferase 2	-1.793
MLXIPL	MLX interacting protein-like	-1.915
MTMR7	myotubularin related protein 7	1.624
NAMPT	nicotinamide phosphoribosyltransferase	-1.576
NPPA	natriuretic peptide A	3.233
Nppb	natriuretic peptide type B	1.520
NUDT7	nudix (nucleoside diphosphate linked moiety X)-type motif 7	-1.522
PCK1	phosphoenolpyruvate carboxykinase 1 (soluble)	-2.481
PCSK9	proprotein convertase subtilisin/kexin type 9	-1.543
PDE8B	phosphodiesterase 8B	1.592
PDPN	podoplanin	1.868
PLA1A	phospholipase A1 member A	-1.824
PLA2G16	phospholipase A2, group XVI	-1.964
PLIN2	perilipin 2	-1.578
PNPLA2	patatin-like phospholipase domain containing 2	-2.022
PNPLA3	patatin-like phospholipase domain containing 3	-2.172
PPARGC1A	peroxisome proliferator-activated receptor gamma, coactivator 1 alpha	-2.164
PPARGC1B	peroxisome proliferator-activated receptor gamma, coactivator 1 beta	-1.702
PRKAR2B	protein kinase, cAMP-dependent, regulatory, type II, beta	-1.709
PRLR	prolactin receptor	-2.029
PTGDS	prostaglandin D2 synthase 21kDa (brain)	-1.914
PTGIS	prostaglandin I2 (prostacyclin) synthase	1.506
RARRES2	retinoic acid receptor responder (tazarotene induced) 2	-2.419
RBP4	retinol binding protein 4, plasma	-2.658

Supplement Table 1: Down-regulation of energy production and lipid metabolism genes in Fst KO MEFs

Symbol	Entrez Gene Name	Fst KO vs WT (Fold Change)
RDH12	retinol dehydrogenase 12 (all-trans/9-cis/11-cis)	-2.360
RETN	resistin	-1.784
SCAP	SREBF chaperone	1.500
SCD	stearoyl-CoA desaturase (delta-9-desaturase)	-1.773
SCHIP1	schwannomin interacting protein 1	1.671
SERINC2	serine incorporator 2	2.085
SERPINA6	serpin peptidase inhibitor, clade A (alpha-1 antiproteinase, antitrypsin), member 6	-1.790
SLPI	secretory leukocyte peptidase inhibitor	-1.824
SNCA	synuclein, alpha (non A4 component of amyloid precursor)	1.595
STAR	steroidogenic acute regulatory protein	-1.808
STARD4	StAR-related lipid transfer (START) domain containing 4	-1.567
SULT1A1	sulfotransferase family, cytosolic, 1A, phenol-preferring, member 1	-1.627
TLR4	toll-like receptor 4	-1.546
TNXB	tenascin XB	-1.936
TTPA	tocopherol (alpha) transfer protein	-2.235
TTR	transthyretin	-2.060
UCP1	uncoupling protein 1 (mitochondrial, proton carrier)	-2.850
UCP2	uncoupling protein 2 (mitochondrial, proton carrier)	-2.255
UGT1A1	UDP glucuronosyltransferase 1 family, polypeptide A1	-1.555
UGT2B10	UDP glucuronosyltransferase 2 family, polypeptide B10	-1.730
VTN	vitronectin	-2.332
XDH	xanthine dehydrogenase	-2.285

Supplemental Table 1. Down-regulation of energy production and lipid metabolism related genes in differentiating Fst KO MEF cultures compared to the WT group. Cells were allowed to differentiate under BAT-specific condition as described in Material and Methods and Affymetrix gene expression analysis was performed. Changes in energy production and lipid metabolism specific genes that were above 1.5 fold were selected.
