# Supplementary material for: Follistatin promotes adipocyte differentiation, browning, and energy metabolism
Source: J Lipid Res. 2014 Mar;55(3):375–84. doi: 10.1194/jlr.M039719 (PMC3934723; doi:10.1194/jlr.M039719)
Supplement: Supplemental Data [file supp_M039719_jlr.M039719-2.pdf]

Supplemental Figure 1

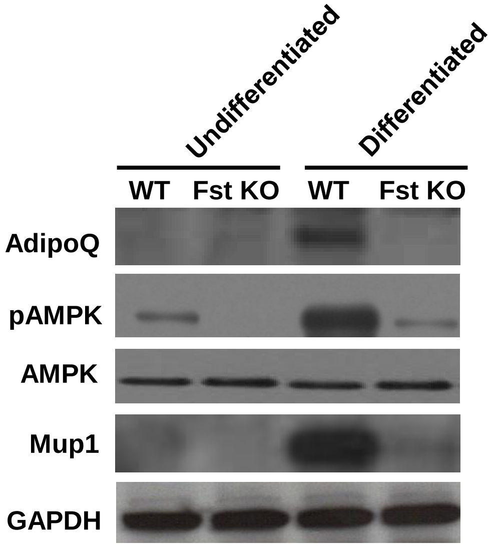

Figure Legend:

Supplemental Figure 1. Western blot analysis of adiponectin (AdipoQ), AMPK/pAMPK and Mup1 in primary cultures of WT and Fst KO MEFs grown either under normal growth medium (undifferentiated) or in modified adipogenic differentiation condition (differentiation). Experiments were conducted in triplicates and a representative western blot is shown.
